# Structural basis of redox-dependent substrate binding of protein disulfide isomerase

**DOI:** 10.1038/srep13909

**Published:** 2015-09-09

**Authors:** Maho Yagi-Utsumi, Tadashi Satoh, Koichi Kato

**Affiliations:** 1 Okazaki Institute for Integrative Bioscience and Institute for Molecular Science, National Institutes of Natural Sciences, 5-1 Higashiyama, Myodaiji, Okazaki, Aichi 444-8787, Japan; 2 Graduate School of Pharmaceutical Sciences, Nagoya City University, 3-1 Tanabe-dori, Mizuho-ku, Nagoya 467-8603, Japan; 3 JST, PRESTO, 3-1 Tanabe-dori, Mizuho-ku, Nagoya 467-8603, Japan

## Abstract

Protein disulfide isomerase (PDI) is a multidomain enzyme, operating as an essential folding catalyst, in which the *b*′ and *a*′ domains provide substrate binding sites and undergo an open–closed domain rearrangement depending on the redox states of the *a*′ domain. Despite the long research history of this enzyme, three-dimensional structural data remain unavailable for its ligand-binding mode. Here we characterize PDI substrate recognition using α-synuclein (αSN) as the model ligand. Our nuclear magnetic resonance (NMR) data revealed that the substrate-binding domains of PDI captured the αSN segment Val37–Val40 only in the oxidized form. Furthermore, we determined the crystal structure of an oxidized form of the *b*′–*a*′ domains in complex with an undecapeptide corresponding to this segment. The peptide-binding mode observed in the crystal structure with NMR validation, was characterized by hydrophobic interactions on the *b*′ domain in an open conformation. Comparison with the previously reported crystal structure indicates that the *a*′ domain partially masks the binding surface of the *b*′ domain, causing steric hindrance against the peptide in the reduced form of the *b*′–*a*′ domains that exhibits a closed conformation. These findings provide a structural basis for the mechanism underlying the redox-dependent substrate binding of PDI.

In the endoplasmic reticulum (ER) of eukaryotic cells, a number of molecular chaperones and folding enzymes assist the proper folding of newly synthesized polypeptide chains. Protein disulfide isomerase (PDI) is a major ER protein that operates as a molecular chaperone and a folding enzyme by catalyzing the formation, cleavage, and rearrangement of the disulfide bonds of unfolded or misfolded proteins^1^,^2^,^3^. After the first description of its enzymatic activity in 1963^4^, extensive structural and functional studies of PDI have been reported using PDI from various species, including human^5^,^6^,^7^,^8^,^9^, yeast^10^,^11^, and thermophilic fungus^12^,^13^,^14^. PDI consists of four tandem thioredoxin-like domains *a*, *b*, *b*′, and *a*′ plus a C-terminal extension^1^,^2^,^3^,^15^, which are arranged into a U-shaped structure^9^,^10^,^11^. Among the four domains, *a* and *a*′ possess a catalytic CXXC motif, which is not shared by *b* and *b*′. Cumulative biochemical data indicate that the *b*′ and *a*′ domains are primarily responsible for substrate recognition^3^,^13^,^16^. In particular, mutational and cross-linking analyses indicate that the *b*′ domain provides the principal peptide binding site in PDI^16^. The *a*′ domain is oxidized by the flavoprotein Ero1 and thereby acts as a disulfide donor for the PDI substrates^17^,^18^.

One unique property of this modular enzyme is that it undergoes conformational rearrangement of the *b*′–*a*′ domains depending on the redox states of the *a*′ active site^13^,^14^. These two domains exhibit a closed conformation in the reduced form and are converted into an open conformation with the exposure of the hydrophobic surface upon oxidation of the *a*′ domain. This conformational transition is supposed to be associated with the redox-dependent substrate binding of PDI. However, no three-dimensional structural data have yet been reported for PDI ligand binding despite the long history of research on this topic. In view of this situation, we attempted to provide the structural basis of PDI substrate recognition by using an appropriate model ligand.

It has been reported that molecular chaperones actively contribute to the suppression of toxic aggregate formation of various amyloidogenic proteins associated with neurodegenerative disorders^19^,^20^. In particular, PDI is upregulated in the brain of patients with Parkinson disease and is found in Lewy bodies^21^, which are composed of the protein α-synuclein (αSN), an intrinsically unstructured protein consisting of 140 amino acid residues associated with other proteins. The increased expression of PDI was also observed in αSN transgenic mice^22^. Moreover, we have recently shown that αSN is capable of interacting with the bacterial chaperone GroEL^23^ and archaeal chaperone PbaB^24^, serving as a useful probe for characterizing their molecular recognition by biophysical techniques, which include nuclear magnetic resonance (NMR) spectroscopy and small-angle neutron scattering. Hence, we undertook to examine the possible interaction of PDI with αSN and, based on the results, we executed structural analyses using X-ray crystallography in conjunction with NMR spectroscopy that focused on the substrate-binding *b*′–*a*′ domains of PDI.

## Results

### Redox-dependent interaction of PDI with αSN

**Figure 1:**
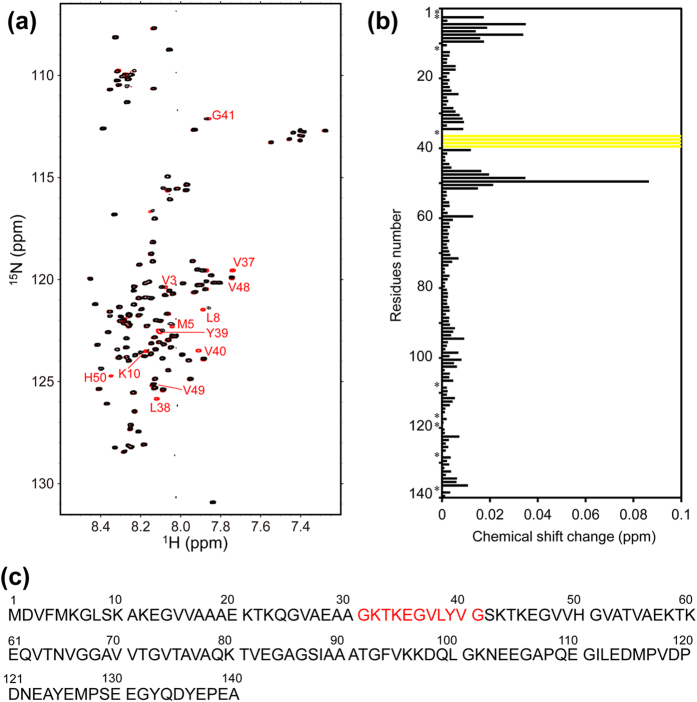
Summary of NMR spectral changes of αSN upon interaction with the oxidized PDI *b*′–*a*′ domains. (**a**) ^1^H-^15^N HSQC spectra of uniformly ^15^N-labeled αSN alone (red) in the presence of the oxidized PDI *b*′–*a*′ domains (black) at a 1:1 molar ratio. (**b**) Plots of the chemical shift changes of the backbone amide peaks of αSN upon interaction with the oxidized PDI *b*′–*a*′ domains. Yellow bars indicate residues whose NMR peaks became undetectable due to extreme broadening upon addition of the PDI *b*′–*a*′ domains. (**c**) The αSN sequence indicates the residues corresponding to the PDI-binding peptide used in the experiments.

To investigate whether αSN can bind PDI, we performed NMR analyses assisted by stable isotope labeling. We prepared ^15^N-labeled αSN and observed the heteronuclear single-quantum correlation (HSQC) spectral changes induced upon addition of the PDI *b*′–*a*′ domains. The results indicate that the oxidized *b*′–*a*′ domains caused significant perturbations in the HSQC peaks originating from the αSN segments Val37–Val40 and Val48–Gly51 (Fig. 1a,b), both of which contain hydrophobic (Hb) and aromatic (φ) residues as Hb−Hb−φ triplets (Fig. 1c). Remarkably, the peaks from the former segment almost completely disappeared, indicating its extensive involvement in an interaction with the PDI *b*′–*a*′ domains. On the basis of these data, we concluded that αSN is capable of interacting with PDI through its specific hydrophobic segment. Based on peak intensity attenuation observed upon titration with the *b*′–*a*′ domains, their association constant was estimated as 3 × 10^4^ M^−1^.

**Figure 2:**
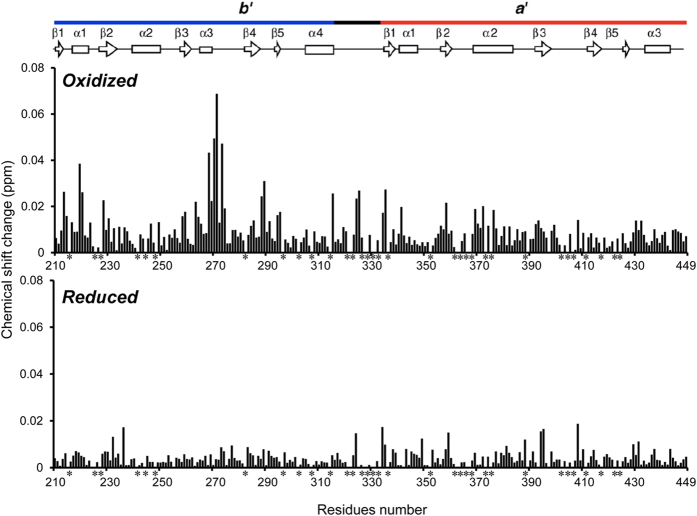
Redox-dependent interaction of the PDI *b*′–*a*′ domains with the αSN peptide probed using NMR. Plots of the chemical shift changes of the backbone amide peaks of the oxidized PDI *b*′–*a*′ domains (upper) or the reduced PDI *b*′–*a*′ domains (lower) upon interaction with the αSN peptide.

We confirmed the binding of this segment using ^15^N-labeled PDI *b*′–*a*′ domains and a synthetic αSN peptide, Gly-Lys-Thr-Lys-Glu-Gly-Val-Leu-Tyr-Val-Gly, which corresponds to the principal binding site of αSN (Fig. 1c). HSQC spectral data indicated that the peptide caused chemical shift perturbations largely for Gly268, His270, Ala271, and Asn273 in the *b*′ domain and, to a lesser extent, for their surrounding residues in the same domain and the residues proximal to the *a*′ active site in the oxidized *b*′–*a*′ form (Fig. 2 and Supplemental Fig. S1). Such spectral changes were much less pronounced in the reduced form of the *b*′–*a*′ domains, indicating that peptide binding depends on the redox states of the *a*′ active site. These data are consistent with those previously obtained using mastoparan as the model ligand, which preferentially binds the oxidized form of the *b*′–*a*′ domains^13^. The redox-dependent interaction was confirmed between the PDI *b*′–*a*′ domains and full-length αSN (Supplementary Fig. S2).

### Crystal structure of the PDI *b*′–*a*′ domains in complex with the αSN peptide

To determine the interaction mode of PDI with αSN, we carried out X-ray crystallographic analysis using the oxidized form of the PDI *b*′–*a*′ domains and the αSN peptide. We successfully crystallized their complex and determined the crystal structure at 1.60 Å resolution. The final model, refined to a resolution of 1.60 Å, had an *R*_work_ of 18.4% and *R*_free_ of 21.7% (Supplemental Table S1). The crystal belonged to space group *P*2_1_2_1_2_1_ with one *b*′–*a*′ molecule and one αSN peptide per asymmetric unit.

**Figure 3:**
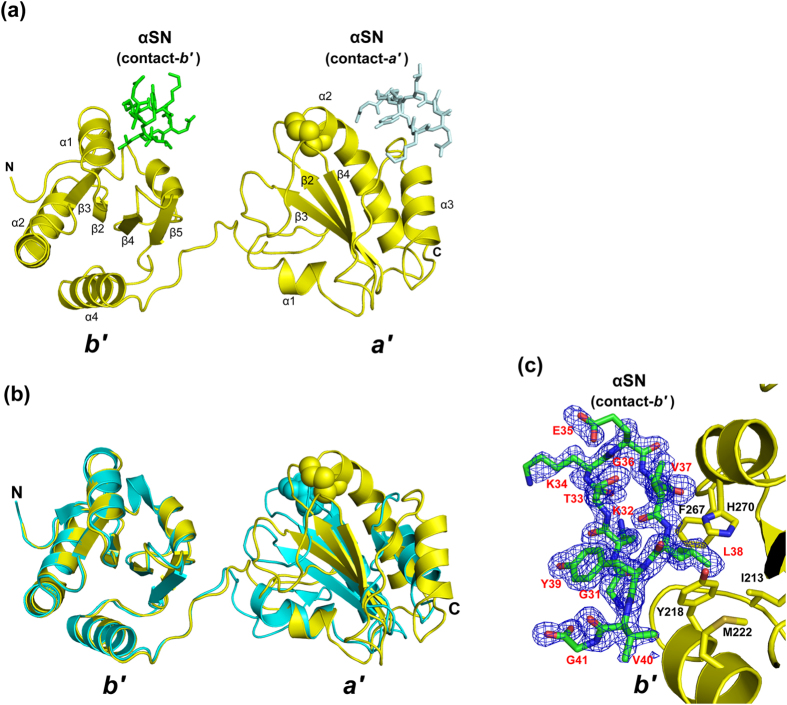
Crystal structure of the oxidized PDI *b*′–*a*′ domains complexed with the αSN peptide. (**a**) Overall view of the PDI *b*′–*a*′/αSN complex. The PDI molecule is yellow, whereas the αSN peptide is green (contact*-b*′) and pale blue (contact*-a*′). The active-site half-cystine residues are shown in sphere models. (**b**) Comparison between the liganded and unliganded PDI *b*′–*a*′ domain. Ribbon models of the liganded (yellow) and unliganded (cyan, PDB code: 3WT2) PDI *b*′–*a*′ domains are shown. (**c**) Close-up view of the contact*-b*′ interface between PDI (yellow) and the αSN peptide (green). Omit *F*_o_-*F*_c_ electron density map of αSN contoured at 2.0 σ.

The PDI *b*′–*a*′ construct we used for crystallization consisted of residues 208–449, and all residues were ordered in the electron density map. Even though the two-domain arrangement was extensively affected by the crystal packing, the *b*′–*a*′ domains showed an open conformation in the oxidized state (Fig. 3a). Due to the crystal packing, the spatial domain arrangement of the αSN-bound oxidized PDI *b*′–*a*′ domains was remarkably different from that of the unliganded form (PDB code: 3WT2)^25^, suggesting the dynamic nature of the interdomain substrate-binding region (Fig. 3b). Each domain structure of the complexed form was essentially identical to those of the apo form with the RMSD of 0.41 and 0.35 Å for the *b*′ and *a*′ domains, respectively. Concerning the bound αSN undecapeptide, all residues were clearly visible in the electron density map (Fig. 3c). Interestingly, the αSN undecapeptide adapts a β-hairpin structure in the crystal.

Because a crystallographically neighboring molecule was accommodated in contact with the two domains, two different interaction modes were observed between the PDI *b*′–*a*′ domains and the αSN peptide (Fig. 3a). One interaction mode (termed contact*-b*′) was mediated through the *b*′ domain surface proximal to the *a*′ domain with a peptide-binding area of 391.6 Å^2^. The other interaction mode (termed contact*-a*′) gave a smaller interface area with 242.9 Å^2^ exclusively on the *a*′ domain. In contact*-b*′, the αSN peptide was recognized through several hydrophobic interactions involving Leu38 and Val40 of αSN and Ile213, Tyr218, Met222, and Phe267 of PDI (Fig. 3c). Furthermore, the main-chain amide group of Leu38 makes a hydrogen bond with His270 Nδ1 atom. In contact*-a*′, in addition to the hydrophobic interactions mediated by Tyr39^αSN^, the peptide ligand was recognized through electrostatic interactions between the C-terminal carboxyl group of Gly41^αSN^ and Arg431^PDI^ (Supplemental Fig. S3). The extent of the interface area and the number of intermolecular interactions suggest that contact*-b*′, rather than contact*-a*′, primarily mediates the interaction.

**Figure 4:**
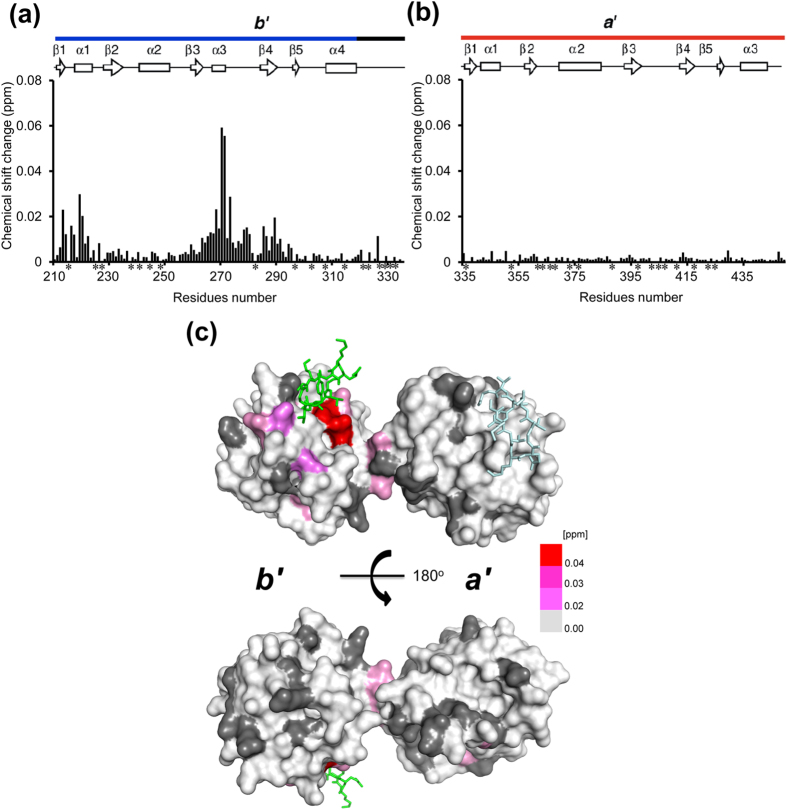
The *b*′ domain of PDI provides the principal binding site for αSN. Plots of the chemical shift changes of the backbone amide peaks of uniformly ^15^N-labeled PDI *b*′ domain (**a**) and oxidized PDI *a*′ domain (**b**) upon interaction with αSN peptide. Proline residues and the residues whose ^1^H-^15^N HSQC peaks could not be observed because of peak overlapping and/or broadening are shown by asterisks. (**c**) Mapping on the crystal structure of the oxidized PDI *b*′–*a*′ domains of residues exhibited chemical shift perturbations [(0.04Δ*δ*_N_^2^ + Δ*δ*_H_^2^)^1/2^ > 0.02 ppm] upon addition of 4 molar equivalent of αSN peptide in the oxidized PDI *b*′–*a*′ domains. The red gradient indicates the strength of the perturbation. The proline residues and the residues whose ^1^H-^15^N HSQC peaks could not be observed as probe because of broadening and/or overlapping are shown in gray.

To probe the peptide binding sites in solution, we examined possible spectral changes of isolated *b*′ and *a*′ domains upon addition of the αSN peptide. The results indicated that the *b*′ but not the *a*′ domain exhibited extensive chemical shift perturbations, consistent with observations of the connected *b*′–*a*′ domains (Fig. 4). These data clearly indicate that the *b*′ domain provides the principal binding site of the hydrophobic segment of αSN.

## Discussion

In the present study, we found that PDI can capture the hydrophobic segment of αSN primarily through its *b*′ domain and determined their binding mode in detail. The hydrophobic PDI-binding segment identified herein is also involved in interactions with GroEL^23^ and PbaB^24^, suggesting that it displays a *chaperone-philic* binding motif that can be widely recognized as a mimic of the malfolded protein hallmarks. Hence, the αSN peptide employed in this study would offer a useful tool for probing chaperone interactions because of its potential broad reactivity with various molecular chaperones.

The αSN peptide contact site largely overlaps with the *b*′ surface involved in interactions with somatostatin and mastoparan, peptide inhibitors that compete with substrates, and with hydrophobic fluorescent probe ANS, which was previously characterized by NMR chemical shift perturbation experiments^3^,^5^,^13^. The present crystal structure successfully provides an atomic view of the molecular recognition of the substrate-binding site of PDI, which is primarily characterized by hydrophobic interactions (Fig. 3).

**Figure 5:**
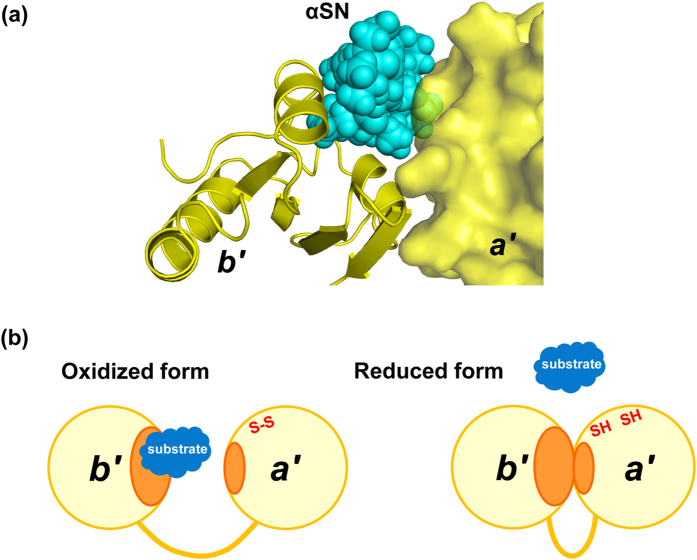
Working mechanism of substrate-binding of PDI. (**a**) Model of closed form of fungal PDI *b*′–*a*′ domains based on the fungal PDI/αSN complex superimposed on the crystal structure of human PDI (PDB code: 3UEM). The *b*′ and *a*′ domains are shown as ribbon and surface representations, respectively, while the αSN peptide is as cyan spheres. (**b**) Schematic model of the redox-dependent substrate binding of PDI. PDI captures the hydrophobic segments of substrates through its hydrophobic surface (orange) exposed in the open conformation of the *b*′–*a*′ domains in its oxidized form, while reduction of the *a*′ active site is coupled with the interdomain *b*′–*a*′ interaction, resulting in release of the substrate with disulfide formation.

Our previous small-angle X-ray scattering data demonstrated that reduced-state PDI *b*′–*a*′ domains adopt a closed conformation in which the hydrophobic ligand binding surface is supposed to be shielded from the solvent^13^,^14^. The crystal structure with a closed conformation of the *b*′–*a*′ domains has been available only for human PDI with the reduced *a*′ active site^8^,^9^. Our structural model based on this crystal structure indicates that the *a*′ domain masks parts of the ligand binding surface of *b*′ and causes steric hindrance, with the αSN peptide accommodated on the *b*′ domain, which results in impaired interaction with the peptide in the closed conformation (Fig. 5a). This explains why this peptide preferentially binds the oxidized form of the PDI *b*′–*a*′ domains (Fig. 5b). In this crystal structure, the peptide was stabilized in the compact β-hairpin conformation due to the crystal contacts. However, physiological substrates of PDI are generally more bulky and mobile in solution and therefore would cause more substantial steric clashes.

In summary, this study presents the first crystallographic snapshot of presumably dynamic PDI interactions with ligand peptides. Our findings provide a structural basis for the mechanisms underlying the redox-dependent substrate binding of PDI, which captures the hydrophobic segments of substrates through its hydrophobic surface that is exposed in the open conformation of the *b*′–*a*′ domains in its oxidized form. Reduction of the *a*′ active site is coupled with the interdomain *b*′–*a*′ interaction, resulting in release of the substrate with disulfide formation.

## Methods

### Protein expression and purification

Expression and purification of the PDI *b*′–*a*′ domains (residues 208–449), *b*′ domain (residues 208–335), and *a*′ domain (residues 334–449) from *Humicola insolens* were performed as previously described^12^,^13^,^25^. To prepare the oxidized form, the purified protein (1 mg/ml) was dialyzed against 50 mM Tris-HCl (pH 8.0) containing 0.1 mM oxidized glutathione for a week. To prepare the reduced form, the protein was dissolved in a buffer containing 10 mM dithiothreitol (DTT). The expression and purification of ^15^N-labeled αSN were performed as previously described^26^. Synthetic αSN peptide (Gly-Lys-Thr-Lys-Glu-Gly-Val-Leu-Tyr-Val-Gly) was purchased from Wako Pure Chemical Industries, Ltd.

### NMR measurements and analyses

NMR measurements were performed in 10 mM sodium phosphate buffer (pH 7.0) containing 100 mM KCl, and 10% (v/v) D_2_O using an AVANCE800 spectrometer (Bruker Biospin) equipped with a 5 mm triple-resonance cryogenic probe. To prepare the reduced form of the PDI proteins, 10 mM d-DTT was added to the buffer. The ^1^H-^15^N HSQC spectra were recorded at a ^1^H observation frequency of 800.32 MHz with 256 (*t*_1_) × 2048 (*t*_2_) complex points. The spectral data of the PDI-derived proteins (at a concentration of 0.05 mM) were acquired at 303 K in the presence and absence of 0.2 mM full-length αSN or 0.2 mM αSN peptide. The spectral assignments of the PDI *b*′–*a*′ domains, *b*′ domain, and *a*′ domain have been described previously^12^. The HSQC spectra of ^15^N-labeled full-length αSN (at a concentration of 0.05 mM) were measured at 283 K in the presence and absence of 0.01–0.05 mM PDI *b*′–*a*′ domains. The NMR assignments of αSN have been described previously^26^. Chemical shift perturbations were quantified as (0.04Δ*δ*_N_^2^ + Δ*δ*_H_^2^)^1/2^, where Δ*δ*_H_ and Δ*δ*_N_ are the observed chemical shift changes for ^1^H and ^15^N, respectively. The NMR data were processed and analyzed using TOPSPIN-2.1 (Bruker Biospin) and SPARKY^27^ software. In NMR perturbation profiles, proline residues and the residues whose ^1^H-^15^N HSQC peaks could not be observed because of peak overlapping and/or broadening were shown by asterisks.

### Protein crystallization, X-ray data collection, and structure determination

The crystals of the PDI *b*′–*a*′ domains (10 mg/ml) complexed with αSN peptide (1:5 molar ratio) were grown in 0.1 M HEPES buffer (pH 7.5) containing 25% (w/v) PEG3350 for a week at 293 K. The crystals were directly transferred into the reservoir solution and flash-cooled in liquid nitrogen. The diffraction data set was collected using synchrotron radiation at BL44XU of SPring-8 (Japan), and was scaled and integrated using HKL2000^28^. Crystal parameters are summarized in Supplemental Table S1.

The 1.60-Å resolution crystal structure of the PDI *b*′–*a*′ domains complexed with the αSN peptide was solved by molecular replacement using the program MOLREP^29^ with the isolated *b*′ and *a*′ domain coordinates derived from the crystal structure of *H. insolens* PDI *b*′–*a*′ domain (oxidized form, 3WT2)^25^ as search models. Model building into the electron density maps and refinement were performed using COOT^30^ and REFMAC5^31^, respectively. The stereochemical quality of the final model was validated by PROCHECK^32^. The final refinement statistics are summarized in Supplemental Table S1. Molecular graphic figures were prepared using PyMOL (http://www.pymol.org/).

## Additional Information

**Accession codes:** The coordinate and structural factor of the crystal structure of the PDI *b′–a′* domains complexed with αSN peptide has been deposited in the Protein Data Bank under the accession numbers 5CRW.

**How to cite this article**: Yagi-Utsumi, M. *et al.* Structural basis of redox-dependent substrate binding of protein disulfide isomerase. *Sci. Rep.* **5**, 13909; doi: 10.1038/srep13909 (2015).

## Supplementary Material

Supplementary Information
