# Supplementary material for: Structural basis of redox-dependent substrate binding of protein disulfide isomerase
Source: Sci Rep. 2015 Sep 9;5:13909. doi: 10.1038/srep13909 (PMC4563560; doi:10.1038/srep13909)
Supplement: Supplementary Information [file srep13909-s1.pdf]

Supplementary information

Structural basis of redox-dependent substrate binding of protein disulfide isomerase

Maho Yagi-Utsumi[1,2], Tadashi Satoh[2,3], and Koichi Kato[1,2*]

[1]Okazaki Institute for Integrative Bioscience and Institute for Molecular Science, National Institutes of Natural Sciences, 5-1 Higashiyama, Myodaiji, Okazaki, Aichi 444-8787, Japan; [2]Graduate School of Pharmaceutical Sciences, Nagoya City University, 3-1 Tanabe-dori, Mizuho-ku, Nagoya 467-8603, Japan; [3]JST, PRESTO, 3-1 Tanabe-dori, Mizuho-ku, Nagoya 467-8603, Japan

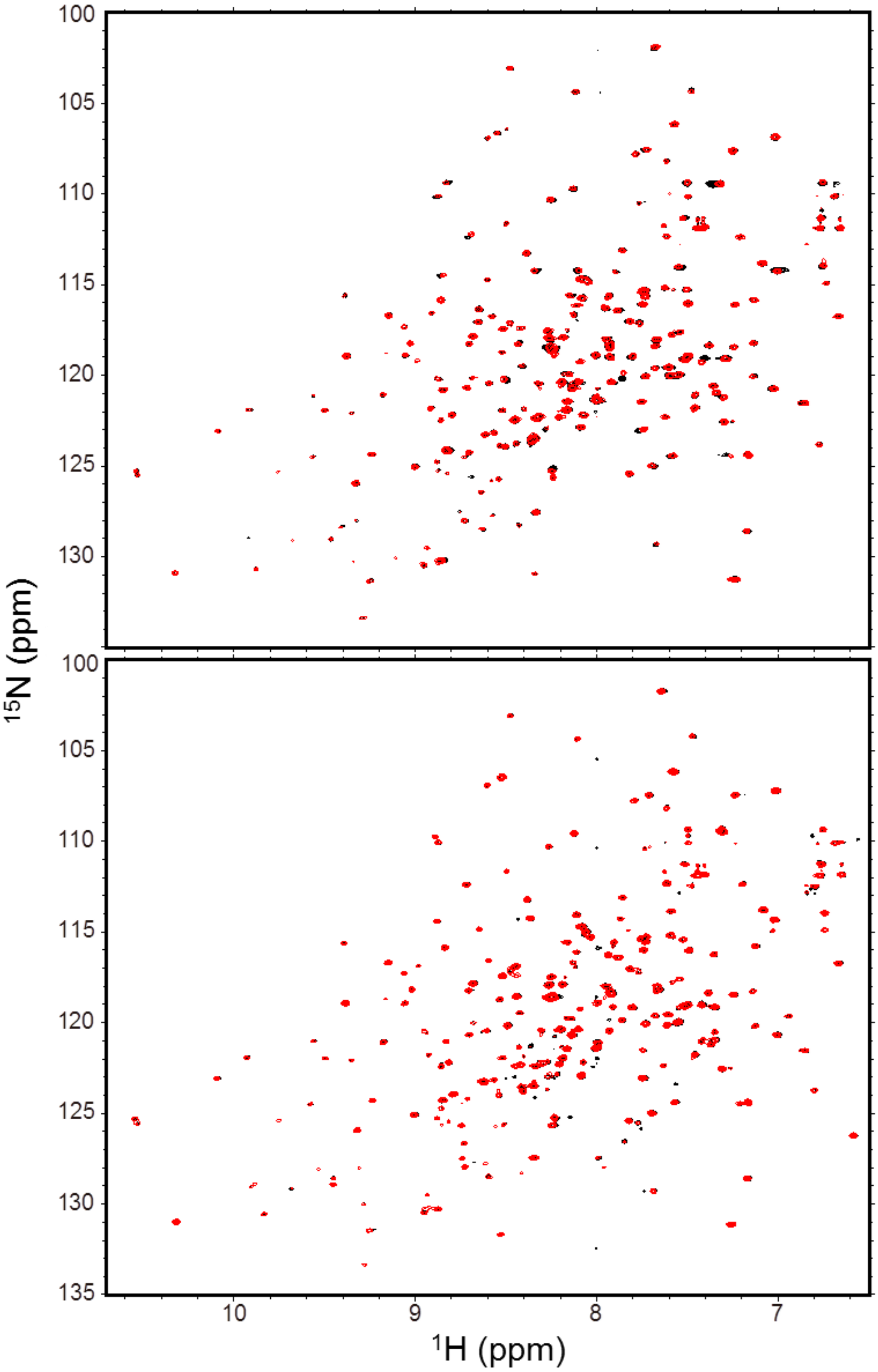

Fig. S1. Redox-dependent interaction of the PDI *b′–a′* domains with the αSN peptide probed using NMR Superposition of ^{1}H-^{15}N HSQC spectra of uniformly ^{15}N-labeled oxidized (upper) and reduced (lower) PDI *b′–a′* domains in the absence (black) and presence (red) of the αSN peptide at a 1:4 molar ratio of PDI *b′–a′* to the αSN peptide.

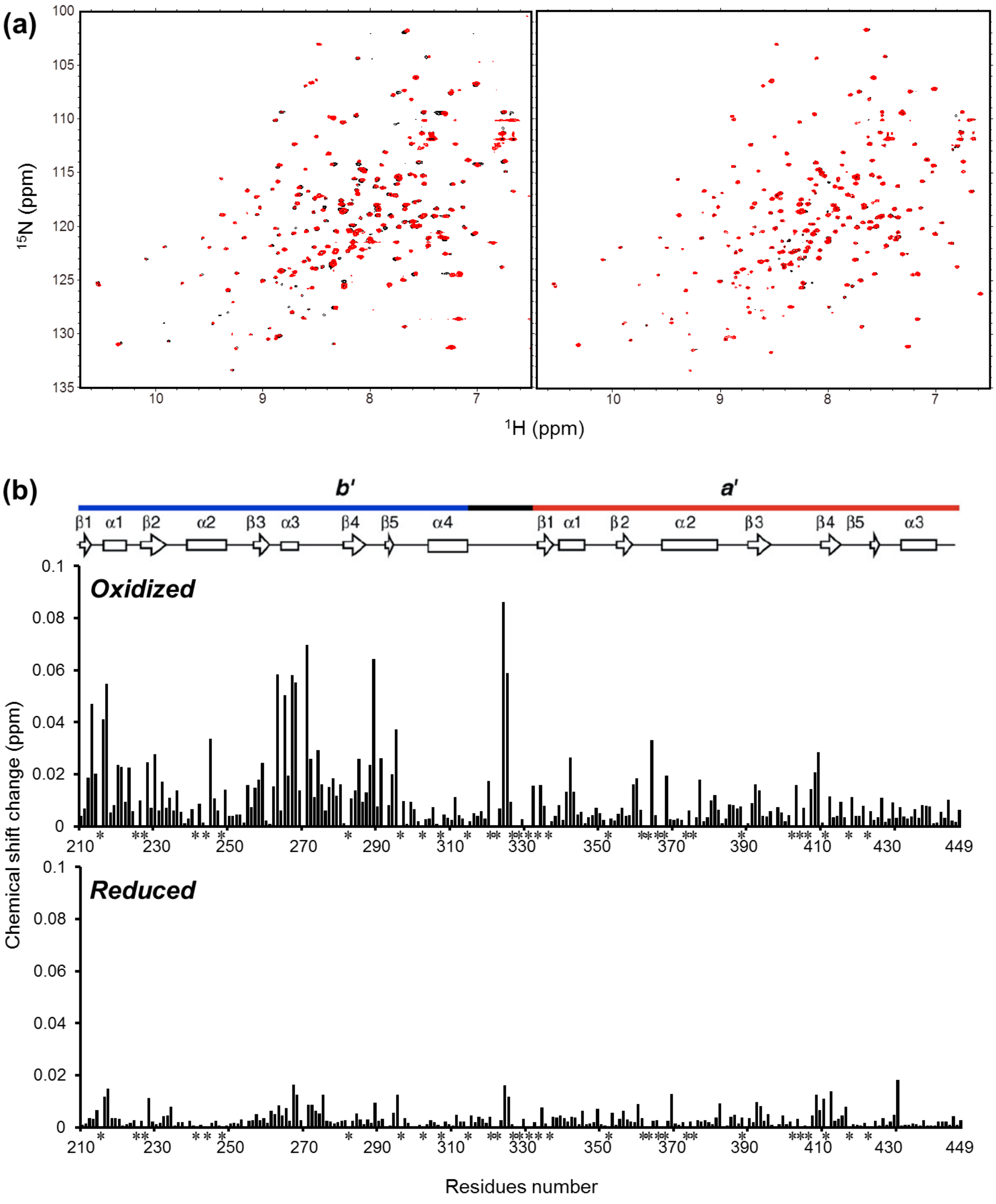

Fig. S2. Redox-dependent interaction of the PDI *b′–a′* domains with αSN probed using NMR (a) Superposition of ^{1}H-^{15}N HSQC spectra of uniformly ^{15}N-labeled oxidized (left) and reduced (right) PDI *b′–a′* domains in the absence (black) and presence (red) of αSN at a 1:4 molar ratio of PDI *b′–a′* domains to αSN. (b) Plots of the chemical shift changes of the backbone amide peaks of the oxidized PDI *b′–a′* domains (upper) or the reduced PDI *b′–a′* domains (lower) upon interaction with αSN.

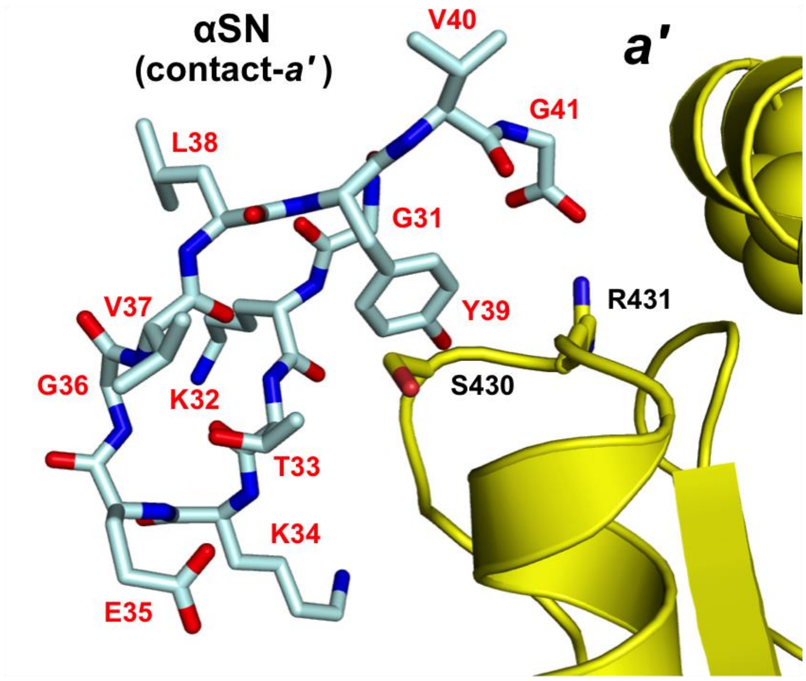

Fig. S3. Close-up view of the contact-*a′* interface between PDI (yellow) and the αSN peptide (pale blue)

Table S1. Data collection and refinement statistics for PDI *b′-a′* domains complexed with αSN peptide.

		PDI *b′-a′*/αSN
**Crystallographic data**		
Space group		$P2_12_12_1$
Unit cell	*a*/*b*/*c* (Å)	58.3/62.8/68.4
	α/β/γ (°)	90.0/90.0/90.0
**Data processing statistics**		
Beam line		SPring-8 BL44XU
Wavelength (Å)		0.90000
Resolution (Å)		50−1.60 (1.63−1.60)
Total/unique reflections		193982/33940
Completeness (%)		95.6 (97.1)
R_{merge} (%)		6.7 (38.9)
I / σ (I)		50.0 (10.3)
**Refinement statistics**		
Resolution (Å)		20.00−1.60
R_{work} / R_{free} (%)		18.4/21.7
R.m.s. deviations from ideal		
Bond lengths (Å)		0.012
Bond angles (°)		1.53
Ramachandran plot (%)		
Most Favored		93.1
Additionally allowed		6.9
Generously allowed		0
Disallowed		0
Number of atoms		
Protein atoms (A/B)		1879/81
Water molecules		339
Average *B*-values (Å^2)		
Protein atoms (A/B)		26.2/28.9
Water molecules		36.6
